# Betulonic Acid, as One of the Active Components of the *Celastrus orbiculatus* Extract, Inhibits the Invasion and Metastasis of Gastric Cancer Cells by Mediating Cytoskeleton Rearrangement In Vitro

**DOI:** 10.3390/molecules27031025

**Published:** 2022-02-02

**Authors:** Zewen Chu, Yuanyuan Luo, Tengyang Ni, Miao Zhu, Xinyi Feng, Yanqing Liu, Haibo Wang

**Affiliations:** 1 Department of Integrated Chinese and Western Medicine, Institute of Translational Medicine, Medical College, Yangzhou University, Yangzhou 225001, China; 17521176065@163.com (Z.C.); lyy528539840@163.com (Y.L.); yzunitengyang@sina.com (T.N.); realzhumiao@163.com (M.Z.); fengxy9633@163.com (X.F.); 2 The Key Laboratory of Syndrome Differentiation and Treatment of Gastric Cancer of the State Administration of Traditional Chinese Medicine, Department of Integrated Chinese and Western Medicine, Yangzhou University, Yangzhou 225001, China; 3 Department of Integrated Chinese and Western Medicine, Yangzhou Cancer Research Institute, Yangzhou University, Yangzhou 225001, China

**Keywords:** *Celastrus orbiculatus*, EMT, MMP, cytoskeleton rearrangement

## Abstract

Gastric cancer is a type of malignant tumor that seriously threatens human life and health. Invasion and metastasis present difficulties in the treatment of gastric cancer, and the remodeling of the tumor cytoskeleton plays an important role in mediating the ability of tumor cells to achieve invasion and metastasis. Previous experimental results suggest that *Celastrus orbiculatus* extract can regulate cytoskeletal remodeling in gastric cancer, but the active component has not been determined. Betulonic acid, as an effective component of COE, inhibits the invasion and metastasis of gastric cancer cells by regulating cytoskeletal remodeling in vitro; its specific mechanisms have been studied here. After betulonic acid was dissolved, it was diluted to various working concentrations in RPMI-1640 medium and added to AGS, HGC-27 and GES-1 cell lines. Cell viability was assessed by CCK-8 and colony formation assays. Cytoskeleton staining was used to detect changes in cytoskeleton morphology. Functional assays including wound healing assays and transwell assays were used to detect the invasion and migration of cells. The effect of betulonic acid on cell invasion and migration was clearly and precisely observed by high-content imaging technology. Western blotting was used to detect the regulation of matrix metalloproteinase-related proteins and epithelial–mesenchymal transformation-related proteins. We found that betulonic acid inhibited the migration and invasion of gastric cancer cells. Therefore, betulonic acid inhibits the invasion and metastasis of gastric cancer cells by mediating cytoskeletal remodeling and regulating epithelial mesenchymal transformation.

## 1. Introduction

Gastric cancer (GC) is a malignant tumor originating from the gastric mucosa epithelium. In 2020, 1,089,000 new cases and 769,000 deaths from GC were reported worldwide, ranking fifth in incidence and fourth in mortality among all malignant tumors [1]. At present, Chinese herbal medicine has been gradually proven to have anti-tumor effects in experimental or clinical studies. More and more researchers are turning their attention to traditional Chinese medicine and its monomer compounds in order to search for more effective treatments with fewer side effects.

Invasion and metastasis present difficulties in the treatment of GC. The invasion and metastasis of GC cells require a series of changes in biological characteristics, including epithelial–mesenchymal transformation (EMT), pseudopodia formation, and the secretion of invasion and metastasis-related factors and enzymes. The remodeling of the tumor cytoskeleton plays an important role in mediating the invasion and metastasis ability of tumor cells [2,3]. Tumor cytoskeleton remodeling refers to the re-disassembly and assembly of fibrous and spherical microfilaments in the cytoskeleton during the movement of tumor cells, forming a new cytoskeleton structure and changing its morphology (including the formation of pseudopodia), making tumor cells easily able to migrate [4,5]. Tumor cells transform from epithelial cell morphology to mesenchymal cell morphology by remodeling their cytoskeleton, and at the same time, remodeling their skeleton at the cell edge, forming polar lamellar and filamentous pseudopodia structures. The part of the pseudopodia in contact with the matrix is enriched with relevant adhesion molecules such as FAK, etc. Under the action of the cell contraction force, the cell protrudes forward repeatedly, and the cell body keeps circulating forward after pulling—thus completing the invasion and metastasis of cells [6]. It can be concluded that the cytoskeleton remodeling of GC tumors is involved in the connection between GC invasion and metastasis, and the remodeling rate of the GC cytoskeleton directly affects the migration ability of tumor cells, which is the structural basis and precondition for GC invasion and metastasis [7,8]. Meanwhile, EMT is a biological process in which cells acquire the ability to invade and metastasize, and cytoskeletal remodeling is the physical structural basis of EMT [9].

Traditional Chinese medicine (TCM) *Celastrus orbiculatus* belongs to the genus *Celastrus*. As a traditional Chinese medicine, *Celastrus orbiculatus* has the function of dispelling wind, dehumidifying, activating blood circulation, detoxifying and reducing swelling [10]. This research group has obtained a patent authorization for the ethyl acetate extract of the *Celastrus orbiculatus* stem and its preparation and application (Grant No: 200710025343.3). Previous experimental studies have shown that *Celastrus orbiculatus* extract (COE) has obvious anti-tumor effects [11,12,13,14] and can inhibit the invasion and metastasis of gastric cancer cells by regulating cytoskeletal remodeling [15]. However, it is not clear which monomer is responsible for this. In early-stage studies, our research group successfully isolated and identified the effective monomer components in COE, and selected betulonic acid for further investigation according to the initial experimental results [16,17].

This study proposes to use in vitro experiments to investigate whether betulonic acid, on the molecular level, has an inhibitory effect on the invasion and metastasis of GC and its specific mechanisms. This will clarify the role of COE-induced suppression of multiple targets connecting the molecular mechanisms of invasion and metastasis in GC, in order to further develop COE as an anti-tumor drug; the next step is to carry out in vivo tests that will lay a more solid theoretical and experimental foundation.

## 2. Results

### 2.1. Effects of Betulonic Acid on the Viability of AGS, HGC-27 and GES-1 Cells

**Figure 1:**
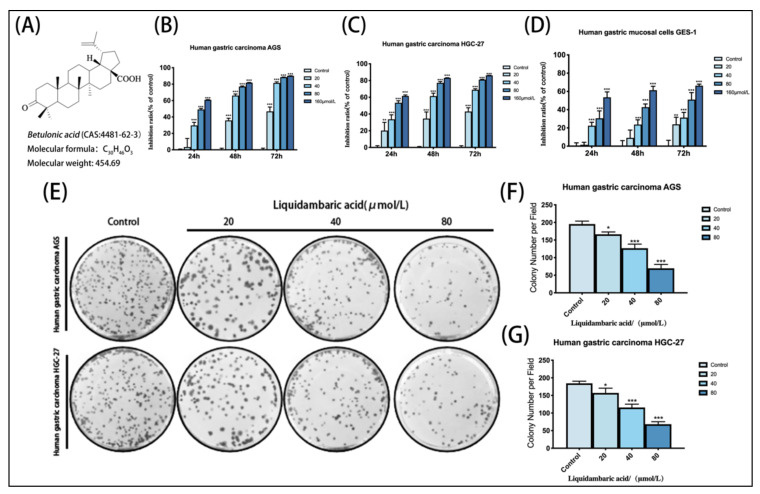
(**A**) Chemical structures of betulonic acid. (**B**) Growth inhibitory effects of betulonic acid on AGS. (**C**) Growth inhibitory effects of betulonic acid on HGC-27. (**D**) Growth inhibitory effects of betulonic acid on GES-1 cells treated with various concentrations of betulonic acid (20, 40, 80 and 160 μM) for 24 h, 48 h and 72 h; cell viability was assessed by CCK-8 assay. (**E**) After the colony formation experiment, cells were fixed and stained with crystal violet solution, and then the results were photographed. (**F**) Statistics of colony formation experiment results of AGS in gastric cancer cells. (**G**) Statistics of colony formation experiment results from HGC-27 gastric cancer cells. * *p* < 0.05, ** *p* < 0.01 and *** *p* < 0.001.

The CCK-8 assay showed that AGS and HGC-27 cells treated with betulonic acid showed concentration- and time-dependent inhibition compared with the control group (Figure 1B,C). At the same time, the IC_50_ values of betulonic acid treatment of AGS, HGC-27 and GES-1 cells over 24 h were calculated according to the experimental results (Table 1). According to the experimental results, the IC_50_ of GES-1 at 24 h, 48 h and 72 h was higher than that of AGS and HGC-27 gastric cancer cells, and the inhibition rate was also lower than that of these two cell lines—indicating that AGS and HGC-27 gastric cancer cells were more sensitive to betulonic acid than gastric GES-1 mucosa cells. In order to exclude the toxic effects of betulonic acid on the cells, the AGS and HGC-27 cells were treated with low concentrations of betulonic acid (20, 40 and 80 μM) for 24 h in the following experiments to investigate the inhibitory effects of betulonic acid on AGS and HGC-27 cell viability. From 24 h, betulonic acid displayed a good inhibitory effect on tumor proliferation as well as demonstrating biological activity, and so 24 h was selected as the time point of study in subsequent experiments. Meanwhile, a colony formation assay also proved that betulonic acid could inhibit the proliferation of gastric cancer cells in a concentration-dependent manner (Figure 1E,F).

### 2.2. Effects of Betulonic Acid on Cytoskeletal Remodeling in Human Gastric Cancer

**Figure 2:**
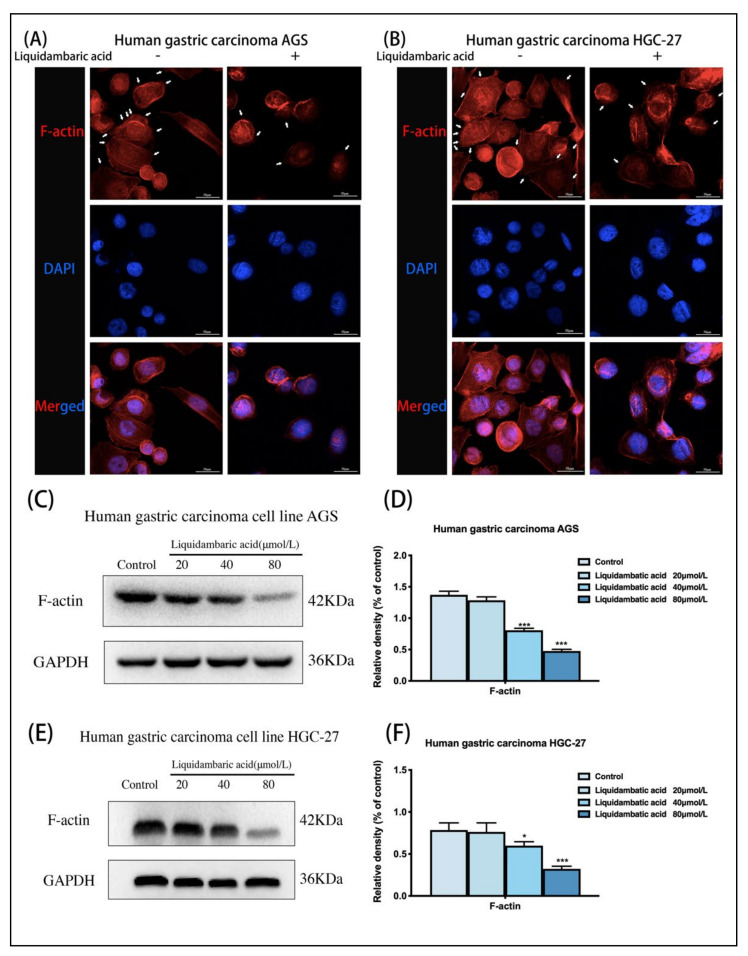
(**A**) Cytoskeleton staining of AGS gastric cancer cells. The white arrows indicate pseudopodia around the cells. Images of cytoskeletal staining were acquired with a confocal laser scanning microscope at 600× magnification. All the scale bars in the figure are 50 μm. (**B**) Cytoskeleton staining of HGC-27gastric cancer cells. The white arrows indicate pseudopodia around the cells. Images of cytoskeletal staining were acquired under confocal laser scanning microscope at 600× magnification. All the scale bars in the figure are 50 μm. (**C**,**D**) Western blot was used to detect the expression of F-actin protein in AGS gastric cancer cells 24 h after betulonic acid treatment. (**E**,**F**) Western blot was used to detect the expression of F-actin protein in gastric cancer cells HGC-27 24 h after betulonic acid treatment. * *p* < 0.05, *** *p* < 0.001.

In order to study the inhibition of betulonic acid on the cytoskeletal remodeling of gastric cancer cells, we conducted cytoskeletal staining experiments; the results showed that the cytoskeletal staining was deeper in the control group, and more pseudopodia were formed around the cells. After 40 μM betulonic acid treatment for 24 h, the cytoskeleton staining was shallow and the number of pseudopodia around the cells decreased. These results suggest that betulonic acid can inhibit cytoskeletal remodeling in gastric cancer (Figure 2A,B).

When we observed cell morphology changes at the microscopic level over time, at changing concentrations of betulonic acid (20 µM, 40 µM, 80 µM), we did not observe that the cells had obvious morphological differences, and were unable to accurately observe any significant differences; as such, we only used a 40 µM dose of betulonic acid to make qualitative observations. The results showed that betulonic acid had an effect on cytoskeletal remodeling. In cytoskeleton staining experiments, we did not perform quantitative studies and did not study the effects of betulonic acid concentration on the cytoskeleton. In the study of the effects of betulonic acid on cytoskeleton proteins, images could not be observed, but Western blots could be used for quantitative study. Therefore, we subsequently conducted Western blot experiments to study the effects of betulonic acid concentration on the protein expression level of the cytoskeleton protein F-actin. Meanwhile, F-actin protein was extracted from two gastric cancer cells for Western blot experiments. AGS and HGC-27 cells treated with betulonic acid for 24 h showed dose-dependent decreases in F-actin expression (Figure 2D,F).

### 2.3. Betulonic Acid Inhibits the Wound-Healing Ability of Gastric Cancer Cells

**Figure 3:**
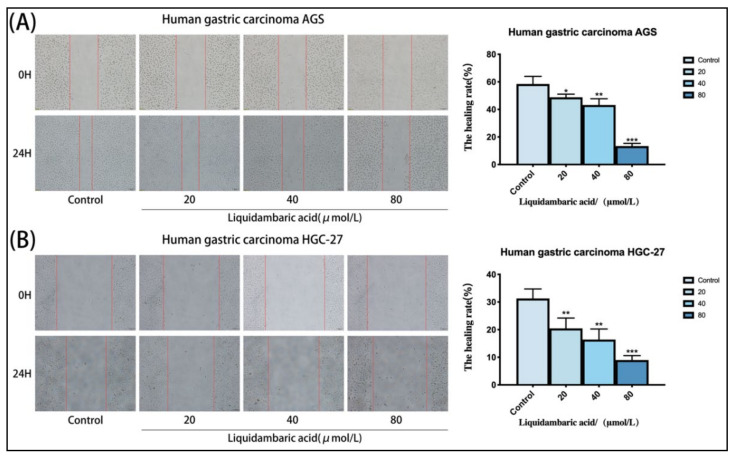
(**A**,**B**) Betulonic acid treatment for 24 h inhibited the migration of AGS cells and HGC-27 cells in a wound-healing assay in a dose-dependent manner, The figure on the right shows the statistical graph of the wound healing rate. Images of AGS and HGC-27 cells were acquired under a microscope at 200× magnification. All the scale bars in the figure are 200 μm. The degree of wound healing (%), calculated as ((scratch width of the control group − scratch width of the betulonic acid group)/scratch width of the control group) × 100%, was used to measure the migration capacity of cells. Scratch width was measured by ImageJ software. * *p* < 0.05, ** *p* < 0.01 and *** *p* < 0.001.

To further study the inhibitory effects of betulonic acid on the invasion and metastasis of gastric cancer cells in vitro, a wound healing experiment was used. The wound healing experiment showed that, compared with the control group, betulonic acid-treated AGS and HGC-27 gastric cancer cells group had increased an wound area and significantly weakened cell-healing ability (Figure 3A,B).

### 2.4. Betulonic Acid Inhibits the Migration and Invasion of Gastric Cancer Cells

**Figure 4:**
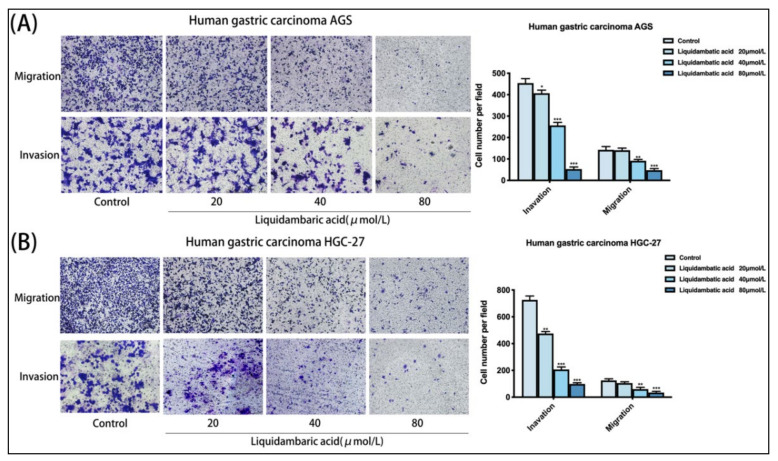
(**A**,**B**) Compared with the control group, betulonic acid-treated AGS and HGC-27 gastric cancer cells had a significantly reduced number of cell-permeable membranes, and the invasion and migration of cells were inhibited. The figure on the right shows the number of transmembrane cells. Images of AGS and HGC-27 cells were acquired under a microscope at 200× magnification. All the scale bars in the figure are 200 μm. * *p* < 0.05, ** *p* < 0.01 and *** *p* < 0.001.

Transwell experiment results showed that, compared with the control group, betulonic acid treatment of AGS and HGC-27 gastric cancer cells significantly reduced the number of cell-permeable membranes, and the invasion and migration of cells were inhibited (Figure 4A,B). The above functional experiments prove that betulonic acid can inhibit the invasion and metastasis of gastric cancer cells in vitro. Images of AGS and HGC-27 cells were acquired under a microscope at 200× magnification.

### 2.5. Dynamic Image of Betulonic Acid Inhibiting the Migration of Gastric Cancer Cells

**Figure 5:**
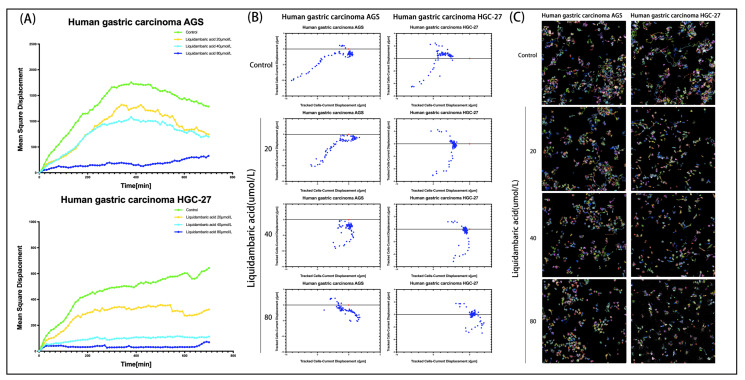
(**A**) Using well-level data, the mean square displacement was plotted against observation time. (**B**) Cell displacement was visualized. Current displacement Y (Y component of a vector from the point of the first to the point of the current observation, averaged over all cells in the well) was plotted against current displacement X (X component of a vector from the point of the first to the point of the current observation, averaged over all cells in the well) using the Multiple Graphs module for display. Each point corresponds to the displacement of a cell at a given time point. (**C**) After treatment with various concentrations of betulonic acid for 12 h, the cells were then placed into the PerkinElmer Operetta CLS High-content Imaging System to continue observing the effects of drug cell migration over 12 h, imaged with the 20× objective in the DPC channel. Cells were identified using the Find Cells module, and migration was monitored using the Track Objects module.

In order to further observe the movement state of cells under the action of betulonic acid, we used PerkinElmer Operetta CLS High-content Imaging System Analysis to visualize cell movement. Analysis with Harmony 4.1 software indicated that the mean square displacement (squared norm of a vector from the point of the first to the point of the current displacement observation, averaged over all cells in the well) of AGS and HGC-27 cells treated with graded concentrations of betulonic acid showed a decreasing trend with increased observation time (Figure 5A). Cell displacement was also visualized, revealing that betulonic acid could inhibit the migration of AGS and HGC-27 gastric cancer cells (Figure 5B). The cells were unlabeled and were imaged in the digital phase contrast (DPC) channel. The DPC image was used to segment the image, and the segmented cells were then tracked. With increases in drug concentration, the migration trajectories became shorter and cell migration was inhibited (Figure 5C).

### 2.6. Effect of Betulonic Acid on the Expression of Epithelial–Mesenchymal Transition (EMT)-Related Proteins in AGS and HGC-27 Cells

**Figure 6:**
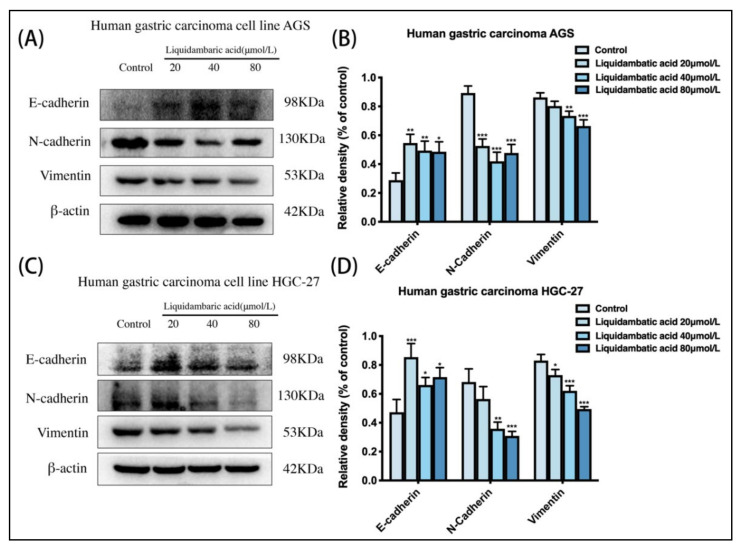
(**A**,**B**) Cell EMT-related protein levels of E-cadherin, N-cadherin, Vimentin and β-actin in AGS and (**C**,**D**) HGC-27 cells were investigated by Western blot. * *p* < 0.05, ** *p* < 0.01 and *** *p* < 0.001.

AGS cells and HGC-27 cells were treated with betulonic acid for 24 h. As shown in Figure 6, the protein expression of E-cadherin increased while that of N-cadherin and Vimentin decreased (Figure 6B,D).

### 2.7. Effect of Betulonic Acid on the Expression of Matrix Metalloproteinase (MMP) Proteins in AGS and HGC-27 Cells

**Figure 7:**
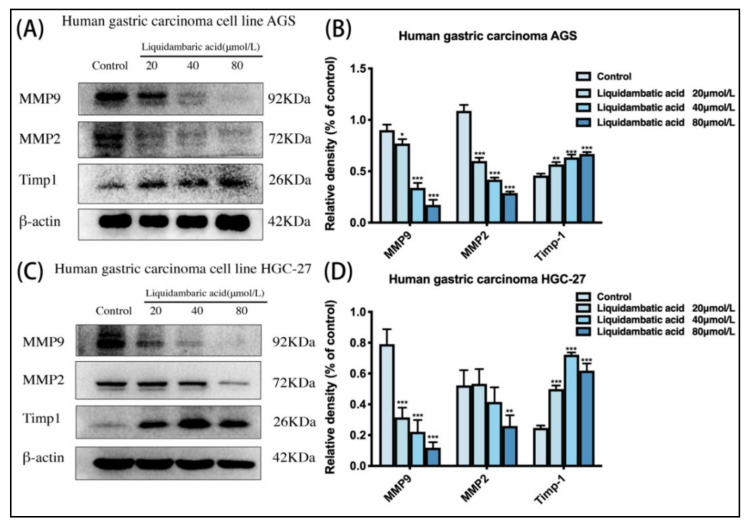
(**A**,**B**) Cell MMP protein levels of MMP-9, MMP-2, Timp1 and β-actin in AGS and (**C**,**D**) HGC-27 cells were investigated by Western blot. * *p* < 0.05, ** *p* < 0.01 and *** *p* < 0.001.

AGS cells and HGC-27 cells were treated with betulonic acid for 24 h. As shown in the Figure 6, the protein expression of Timp1 increased while that of MMP-9 and MMP-2 decreased (Figure 7B,D).

## 3. Discussion

At present, besides surgical resection, lymphatic chemotherapy and molecular targeted therapy, TCM therapy has gradually begun to enter into the lab and clinic due to its advantages of low toxicity and effectiveness. Previous studies have shown that COE (a crude extract of ethyl acetate from the Chinese herbal medicine *Celastrus Orbiculatus*) can inhibit the invasion and metastasis of GC cells by mediating cytoskeletal remodeling [15,18]. In this study, we proved that betulonic acid, as one of the effective components of COE, can also regulate the occurrence of EMT by mediating cytoskeleton remodeling—thus inhibiting the invasion and metastasis of GC cells.

The cytoskeleton is the structural basis for the formation of invasive pseudopodia and platelet pseudopodia, which are the locomotor tools of tumor cell invasion and metastasis [19]. During the invasion and migration of GC cells, the remodeling of the actin cytoskeleton occurs throughout the whole process [20]. Dynamic remodeling of the cytoskeleton (including pseudopodia formation) provides a continuous capacity for cell movement and is an important molecular mechanism for tumor cell invasion and migration [21,22]. In this study, the protein expression level of F-actin was detected by skeletal staining of AGS and HGC-27 GC cells and Western blot assays. The results showed that betulonic acid could mediate cytoskeletal remodeling, inhibit the formation of pseudopodia and regulate the protein expression level of F-actin compared with the control group. This suggests that betulonic acid, as one of the active components of COE, can also mediate cytoskeletal remodeling.

The invasion and metastasis of tumor cells is an important landmark event in solid tumor malignant progression and is an important cause of the low survival rate of GC patients [23]. In the process of tumor invasion and metastasis, tumor cells escape from the primary site to form a single tumor cell and invade the surrounding tissues to break through the tumor barrier, thus entering the blood vessels and spreading to the whole body, and then spilling out of the blood vessels to form distal metastatic lesions [24]. Among these stages, the EMT process is the most critical. The EMT process refers to the close linkage of cells with epithelial polarity, leading to the loss of cell polarity and the formation of a single mesenchymal cell with motility potential [25]. In the process of EMT in GC cells, remodeling of the cytoskeleton is involved in the whole process, providing a physical dynamic basis for EMT. The invasion and migration of a single tumor cell can be roughly divided into five steps: (1) The actin cytoskeleton reorganizes at the front end of the tumor cell to promote the formation of a local convex membrane [26]; (2) Locally raised cell membrane receptors bind to extracellular matrix (ECM) substrates and recruit related molecules to form cell–ECM adhesion [27]; (3) Local convex cells secrete proteolytic ECM substrates into the ECM after adhesion [28]; (4) The intracellular actin cytoskeleton contracts with the mechanical stress provided by adhesion and pulls the cell body forward [29]; (5) Adhesion dissolution of the cell tail and its forward movement with the cell body. In this study, we detected EMT-related markers using Western blotting, and the results showed that betulonic acid could inhibit the EMT process of GC cells by regulating the expression of EMT proteins compared with the control group.

We also detected classic markers of invasion and metastasis—Matrix metalloproteinases (MMPs)—in the cytoplasm of gastric cancer cells, and the results showed that betulonic acid could also affect the expression levels of MMP proteins. Degradation of the extracellular matrix and basement membrane is the most important step in tumor invasion and metastasis. MMPs play an important role in the degradation of the extracellular matrix and basement membrane [30]. Among the MMPs, MMP-2 and MMP-9 are currently the most frequently studied, able to degrade the ECM, greatly change the viscosity and mobility of tumors, and promote invasion and metastasis. The main function of TIMP-1 is its inhibition of MMPs. TIMPs irreversibly inactivate MMPs by directly binding to catalytic zinc cofactors, resulting in protease inhibition [31,32]. In this experiment, we detected the protein expression level of MMPs in the cytoplasm of gastric cancer cells, confirming the effect of betulonic acid on MMP protein levels in gastric cancer cells. Next, we will study the specific mechanisms of betulonic acid inhibiting the invasion and metastasis of gastric cancer from the perspective of MMP activity. A series of functional experiments, such as transwell experiments, wound healing experiments and high-intension imaging, further confirmed that betulonic acid could inhibit the invasion and metastasis of GC cells.

## 4. Materials and Methods

### 4.1. Drug

Betulonic acid (standard substance, HPLC ≥98%) was purchased from Shanghai Yuanye Biotechnology, Shanghai, China (Shanghai Yuanye Biotechnology, Shanghai, China, Cat. no. B20780). Betulonic acid was dissolved in dimethyl sulfoxide (DMSO; Sigma, Berlin, Germany, Cat. no. D2650). The maximum concentration of DMSO in the base solution did not exceed 0.1%. All control subjects (i.e., solvent control subjects) were added to RPMI-1640 medium with DMSO equivalent to the highest dose of betulonic acid.

### 4.2. Reagents

RPMI Medium Modified with 2.05 mM l-Glutamine (HyClone, Waltham, MA, USA, Cat. no. SH30809.01); trypsin, phosphate-buffered saline (PBS; Beyotime Biotechnology, Shanghai, China, Cat. no. C0201, Cat. no. C0221A); fetal bovine serum (FBS; Gibco, Waltham, MA, USA, Cat. no. 10099141); transwell-permeable supports and 8.0 μm polycarbonate membranes (Corning, New York, NY, USA, Cat. no. 356234, Cat. no. 3422); CCK-8 Cell Proliferation and Cytotoxicity Assay Kit (Beijing Solarbio Science & Technology Co., Ltd., Beijing, China, Cat. no. CA1210); tetramethylrhodamine (TRITC)-conjugated phalloidin, an actin staining agent (Merck, Berlin, Germany, Cat. no. FAK100); MMP-2, MMP-9, E-cadherin, N-cadherin, Vimentin and β-actin (Thermo Fisher Scientific, Waltham, MA, USA, Cat. no. PA5-85197, Cat. no. PA5-16509, Cat. no. PA5-32178, Cat. no. PA5-29570, Cat. no. MA5-16409, Cat. no. PA146296); TIMP1, F-actin, GAPDH (Abcam, Cambridge, UK, Cat. no. ab211926, Cat. no. ab130935, Cat. no. ab8245); anti-rabbit IgG, HRP-linked antibody (Cell Signaling Technology, Danvers, MA, USA, Cat. no. 7074).

### 4.3. Cell Culture

The AGS and HGC-27 human gastric cancer cell lines and GES-1 gastric mucosal cell line were purchased from the Procell Life Science & Technology Co., Ltd., Wuhan, China. AGS and HGC-27 cells were cultured in RPMI-1640 medium containing 10% FBS and maintained at 37 °C in a humidified incubator in an atmosphere of 5% CO_2_.

### 4.4. Cell Viability Assay

AGS, HGC-27 and GES-1 cells were plated at 5 × 10^3^ cells/well in 96-well plates (Corning, New York, NY, USA, Cat. no. 3599). Then, the cells were treated with different concentrations of betulonic acid (0, 20, 40, 80, and 160 μM) and incubated overnight, and further cultured for 24 h, 48 h and 72 h. A total of 20 μL CCK-8 solution was added to each well, and incubated for another 4 h. The absorbance (A) value was read at 450 nm using an EnSpire multilabel plate reader (PerkinElmer, Waltham, MA, USA).

### 4.5. Colony Formation Assay

Briefly, 400 cells were seeded in triplicate in 6-well culture plates (Corning, New York, NY, USA, Cat. no. 3516) and allowed to attach for 24 h, followed by undergoing drug treatment for 14 days. To prevent the drying and evaporation of culture media during long-term culture, media were changed and fresh drugs were added in the mid-term of treatment. At the end of the culture, cells were fixed and stained with crystal violet solution. The colonies were counted with ImageJ software (National Institutes of Health, Bethesda, MD, USA).

### 4.6. Cytoskeleton Staining

The cell suspension droplets were placed on sterilized slides, and the slides were mounted in the incubator for 12 h. After drug treatment, they were removed and fixed with 4% formaldehyde. A total of 0.1% Triton X-100 was made up with TRITC-conjugated phalloidin and placed in dark room temperature for 45 min. After incubation with DAPI staining solution, the tablets were sealed with anti-fluorescence quenching sealing solution, and the results were observed and recorded by laser confocal microscopy.

### 4.7. Transwell Chamber Assay

The cells were first added to 30 μL Matrigel gel and solidified at 4 °C. The digested cells were suspended in serum-free medium, and the cell density was adjusted to 1 × 10^5^. The cells were added into the upper Transwell chamber, and betulonic acid was diluted to various working concentrations in medium containing 10% fetal bovine serum, and then added into the lower chamber. After conventional culture at 37 °C for 12–48 h, the cells were removed and wiped with cotton swabs. The cells were washed twice with PBS, fixed with 4% formaldehyde for 15 min, and stained with crystal violet. The number of cells below the membrane was counted randomly under an inverted microscope across 5 fields. The migration test was the same as the invasion test, but Matrigel glue was not required on the inner surface of the membrane at the bottom of the chamber. The number of cells passing through the chamber was analyzed using ImageJ software. Images of AGS and HGC-27 cells were acquired under a microscope at 200× magnification.

### 4.8. Wound Healing Assay

Briefly, cell lines were seeded at a density of 3 × 10^5^ per well in 6-well plates, and linear scratch wounds were created on the confluent cell monolayers using a 200 μL pipette tip; the exfoliated cells were removed by three washes with phosphate-buffered saline (PBS). Images were obtained at 0 h and 24 h. Photographs were taken using an inverted microscope. The degree of wound healing (%), calculated as ((scratch width of the control group − scratch width of the betulonic acid group)/scratch width of the control group) × 100%, was used to measure the migration capacity of cells. Scratch width was measured by ImageJ software. Images of AGS and HGC-27 cells were acquired under a microscope at 200× magnification.

### 4.9. PerkinElmer Operetta CLS High-Content Imaging System Analysis

Briefly, gastric cancer cells were inoculated into 96-well plates at a density of 4 × 10^3^ cells/well and treated overnight with drugs. The plates were then placed into the PerkinElmer CLS high content imaging analysis system for observation for 12 h. Harmony 4.1 software (PerkinElmer, Waltham, MA, USA) was used for data collection and analysis.

### 4.10. Western Blot Analysis

Cells were rinsed with PBS and lysed with RIPA buffer following treatment. The extraction methods of F-actin were as follows: Pre-cooled cracking buffer 1 (50 mM KCl, 100 mM NaF, 10 mM K_2_HPO_4_, 2 mM MgCl_2_, 0.2 mm DTT, 1 mM EGTA, 1 mM sucrose, 0.5% Triton X-100, pH 7.0) was added. All cells were scraped off after ice lysis for 20 min, and centrifuged at 14,000× *g* for 30 min. The supernatant was extracted and precipitated with an equal volume of buffer 2 (20 mM TrIS–HCl, 1 mM sodium acetate, 1.5 mm guanidine hydrochloride, 1 mM CaCl_2_, 1 mM ATP, pH 7.5). The samples were incubated on ice for 1 h and gently stirred every 10 min, and then at 14,000× *g* for 30 min to ensure that F-actin was in the supernatant. After boiling the protein samples in 5 × loading buffer, equal amounts of protein were subjected to sodium dodecyl sulfate-polyacrylamide gel electrophoresis (SDS-PAGE). Separated proteins were transferred onto PVDF membranes. The membranes were blocked with 5% skimmed milk for 2 h and probed with the indicated primary antibodies overnight, including E-cadherin, N-cadherin, Vimentin, MMP-2, MMP-9, β-actin, GAPDH, TIMP1 and F-actin. After washing, the membranes were incubated with primary antibody, followed by anti-rabbit IgG, HRP-linked antibody at room temperature for 2 h. Finally, the membranes were washed extensively and incubated using an ECL chemiluminescent kit (Thermo Fisher Scientific, Waltham, MA, USA, Cat. no. 34580). The blots were visualized by a Gel Doc XR+ System (Bio-Rad, Hercules, CA, USA).

### 4.11. Statistical Analysis

Data are presented as the means ± standard deviation, calculated by one-way analysis of variance (ANOVA) followed by a Bonferroni post-hoc test. * *p* < 0.05 was considered significant. Data analysis was performed using IBM SPSS Statistics 24.0 (IBM, Armonk, NY, USA).

## 5. Conclusions

In conclusion, this study revealed that betulonic acid, as one of the effective active components of COE, has the same anti-invasion and metastasis function on GC cells as COE. Its mechanism may be through the mediation of actin skeleton remodeling in gastric cancer cells, affecting the expression levels of proteins related to the EMT signaling pathway—thus inhibiting the invasion and metastasis of gastric cancer cells. Meanwhile, according to Western blot results, betulonic acid can affect the expression levels of MMP-related proteins in gastric cancer cell lysates. Whether betulonic acid can inhibit the invasion and metastasis of gastric cancer cells by affecting the activity of MMPs is worth further exploration. This study also provided new possible regulatory mechanisms and new research ideas for the identification of the mechanisms of betulonic acid against gastric cancer. In addition, this study will provide a more solid theoretical and experimental basis for further studies, including in vivo experiments. The present study also provides new evidence for the development of COE as a new antitumor drug.

## Figures and Tables

**Table 1:** IC_50_ values of betulonic acid in AGS, HGC-27 and GES-1 cells.

HGC-27 Human Gastric Carcinoma	IC_50_ (μM)		
	24 h	48 h	72 h
	75.06	39.4	33.47
**AGS Human Gastric Carcinoma**	**IC_50_ (μM)**		
	24 h	48 h	72 h
	89.82	38.33	27.43
**GES-1 Human Gastric Mucosal Cells**	**IC_50_ (μM)**		
	24 h	48 h	72 h
	120.84	83.93	73.56

## Data Availability

All data included in this study are available upon request by contact with the corresponding author.

## Sample Availability

Samples of the compounds are available from the authors.

## References

[1] Sung H., Ferlay J., Siegel R.L., Laversanne M., Soerjomataram I., Jemal A., Bray F. (2021). Global Cancer Statistics 2020: GLOBOCAN Estimates of Incidence and Mortality Worldwide for 36 Cancers in 185 Countries. CA Cancer J. Clin..

[2] Li X., Wang J. (2020). Mechanical tumor microenvironment and transduction: Cytoskeleton mediates cancer cell invasion and metastasis. Int. J. Biol. Sci..

[3] Fife C.M., McCarroll J.A., Kavallaris M. (2014). Movers and shakers: Cell cytoskeleton in cancer metastasis. Br. J. Pharmacol..

[4] Huang D., Cao L., Zheng S. (2017). CAPZA1 modulates EMT by regulating actin cytoskeleton remodelling in hepatocellular carcinoma. J. Exp. Clin. Cancer Res..

[5] Zhang S., Wu L., Liu Q., Chen K., Zhang X. (2015). Impact on growth and invasion of gastric cancer cell lines by silencing NEDD9. OncoTargets Ther..

[6] Kazazian K., Go C., Wu H., Brashavitskaya O., Xu R., Dennis J.W., Gingras A.C., Swallow C.J. (2017). Plk4 Promotes Cancer Invasion and Metastasis through Arp2/3 Complex Regulation of the Actin Cytoskeleton. Cancer Res..

[7] Shindo A., Wallingford J.B. (2014). PCP and septins compartmentalize cortical actomyosin to direct collective cell movement. Science.

[8] Xie W., Chen C., Han Z., Huang J., Liu X., Chen H., Zhang T., Chen S., Chen C., Lu M. (2020). CD2AP inhibits metastasis in gastric cancer by promoting cellular adhesion and cytoskeleton assembly. Mol. Carcinog..

[9] Artym V.V., Zhang Y., Seillier-Moiseiwitsch F., Yamada K.M., Mueller S.C. (2006). Dynamic interactions of cortactin and membrane type 1 matrix metalloproteinase at invadopodia: Defining the stages of invadopodia formation and function. Cancer Res..

[10] Guo Y., Li X., Wang J., Xu J., Li N. (2005). A new alkaloid from the fruits of *Celastrus orbiculatus*. Fitoterapia.

[11] Yang L., Liu Y.Q., Wang M., Qian Y.Y., Dai X.J., Zhu Y.D., Chen J., Guo S.Y., Hisamitsu T. (2016). *Celastrus orbiculatus* extract triggers apoptosis and autophagy via PI3K/Akt/mTOR inhibition in human colorectal cancer cells. Oncol. Lett..

[12] Wang H.B., Tao L., Ni T.Y., Gu H., Jin F., Dai X.J., Feng J., Ding Y., Xiao W., Guo S.Y. (2017). Anticancer efficacy of the ethyl acetate extract from the traditional Chinese medicine herb *Celastrus orbiculatus* against human gastric cancer. J. Ethnopharmacol..

[13] Jue C., Min Z., Zhisheng Z., Lin C., Yayun Q., Xuanyi W., Feng J., Haibo W., Youyang S., Tadashi H. (2017). COE inhibits vasculogenic mimicry in hepatocellular carcinoma via suppressing Notch1 signaling. J. Ethnopharmacol..

[14] Zhu Y.D., Liu Y.Q., Qian Y.Y., Zhang H., Li G.Q., Yang L. (2014). Extracts of *Celastrus orbiculatus* exhibit anti-proliferative and anti-invasive effects on human gastric adenocarcinoma cells. Chin. J. Integr. Med..

[15] Wang H.B., Gu H., Feng J., Qian Y.Y., Yang L., Jin F., Wang X.Y., Chen J., Shi Y.Y., Lu S.H. (2017). *Celastrus orbiculatus* extract suppresses the epithelial-mesenchymal transition by mediating cytoskeleton rearrangement via inhibition of the Cofilin 1 signaling pathway in human gastric cancer. Oncol. Lett..

[16] Jiang W., Shan T.Z., Xu J.J., Chen W.J., Miao L., Lv M.Y., Tao L., Liu Y.Q. (2019). Cytotoxic abietane and kaurane diterpenoids from *Celastrus orbiculatus*. J. Nat. Med..

[17] Chu Z.W., Shi X., Chen G.Y., He X.J., Qian Y.Y., Wang H.B., Tao L., Liu Y.Q., Jiang W., Chen J. (2021). COE Inhibits Vasculogenic Mimicry by Targeting EphA2 in Hepatocellular Carcinoma, a Research Based on Proteomics Analysis. Front. Pharmacol..

[18] Wang H.B., Tao L., Jin F., Gu H., Dai X.J., Ni T.Y., Feng J., Ding Y.B., Xiao W.M., Qian Y.Y. (2017). Cofilin 1 induces the epithelial-mesenchymal transition of gastric cancer cells by promoting cytoskeletal rearrangement. Oncotarget.

[19] Yamaguchi H. (2012). [Molecular mechanisms of invadopodium formation by cancer cells]. Seikagaku.

[20] Oser M., Yamaguchi H., Mader C.C., Bravo-Cordero J.J., Arias M., Chen X., Desmarais V., van Rheenen J., Koleske A.J., Condeelis J. (2009). Cortactin regulates cofilin and N-WASp activities to control the stages of invadopodium assembly and maturation. J. Cell Biol..

[21] Greco M.R., Antelmi E., Busco G., Guerra L., Rubino R., Casavola V., Reshkin S.J., Cardone R.A. (2014). Protease activity at invadopodial focal digestive areas is dependent on NHE1-driven acidic pHe. Oncol. Rep..

[22] Saykali B.A., El-Sibai M. (2014). Invadopodia, regulation, and assembly in cancer cell invasion. Cell Commun. Adhes..

[23] Wang N., Liu D., Guo J., Sun Y., Guo T., Zhu X. (2018). Molecular mechanism of Poria cocos combined with oxaliplatin on the inhibition of epithelial-mesenchymal transition in gastric cancer cells. Biomed. Pharmacother..

[24] Iwatsuki M., Mimori K., Yokobori T., Ishi H., Beppu T., Nakamori S., Baba H., Mori M. (2010). Epithelial-mesenchymal transition in cancer development and its clinical significance. Cancer Sci..

[25] Todosi A.M., Gavrilescu M.M., Aniţei G.M., Filip B., Scripcariu V. (2012). Colon cancer at the molecular level--usefulness of epithelial-mesenchymal transition analysis. Rev. Med. Chir. Soc. Med. Nat. Iasi.

[26] Busco G., Cardone R.A., Greco M.R., Bellizzi A., Colella M., Antelmi E., Mancini M.T., Dell’Aquila M.E., Casavola V., Paradiso A. (2010). NHE1 promotes invadopodial ECM proteolysis through acidification of the peri-invadopodial space. FASEB J..

[27] Zeng D., Ferrari A., Ulmer J., Veligodskiy A., Fischer P., Spatz J., Ventikos Y., Poulikakos D., Kroschewski R. (2006). Three-dimensional modeling of mechanical forces in the extracellular matrix during epithelial lumen formation. Biophys. J..

[28] Yamada H., Takeda T., Michiue H., Abe T., Takei K. (2016). Actin bundling by dynamin 2 and cortactin is implicated in cell migration by stabilizing filopodia in human non-small cell lung carcinoma cells. Int. J. Oncol..

[29] Dasgupta S., Cushman I., Kpetemey M., Casey P.J., Vishwanatha J.K. (2011). Prenylated c17orf37 induces filopodia formation to promote cell migration and metastasis. J. Biol. Chem..

[30] Sampieri C.L., León-Córdoba K., Remes-Troche J.M. (2013). Matrix metalloproteinases and their tissue inhibitors in gastric cancer as molecular markers. J. Cancer Res. Ther..

[31] Visse R., Nagase H. (2003). Matrix metalloproteinases and tissue inhibitors of metalloproteinases: Structure, function, and biochemistry. Circ Res..

[32] Nagase H., Visse R., Murphy G. (2006). Structure and function of matrix metalloproteinases and TIMPs. Cardiovasc. Res..

